# Supplementary material for: The comparison of comprehensive sexuality education knowledge and attitudes in CSE-exposed and CSE-naïve non-formal settings in Lagos State, Nigeria
Source: PLOS Glob Public Health. 2024 Oct 31;4(10):e0003858. doi: 10.1371/journal.pgph.0003858 (PMC11527314; doi:10.1371/journal.pgph.0003858)
Supplement: S1 Checklist — (DOCX) [file pgph.0003858.s002.docx]

STROBE Statement—checklist of items that should be included in reports of observational studies

|  | Item No. | Recommendation | Page  No. | Relevant text from manuscript |
| --- | --- | --- | --- | --- |
| **Title and abstract** | 1 | (*a*) Indicate the study’s design with a commonly used term in the title or the abstract | 1 | Comparison of comprehensive sexuality education knowledge and attitudes in CSE-exposed and CSE-naïve non-formal settings in Lagos State, Nigeria. |
|  |  | (*b*) Provide in the abstract an informative and balanced summary of what was done and what was found | 2 | A mixed-method research design was used. The study population comprised of students 15-24 years of age attending technical and vocational centers in Lagos State and instructors in CSE-exposed centres. Sample size of 450 per group was calculated. A structured interviewer-administered questionnaire; focus group discussions and key informant interviews were used for data collection. Quantitative data was analyzed at p<0.05; coding and thematic analysis of qualitative data was followed by integration of the findings; and interpreted using the information-motivation-behavioural skills model. Students in CSE-exposed schools had statistically significantly higher SRHR-related knowledge scores and positive attitudes towards inclusive gender norms. The CSE training improved the educational knowledge and teaching modalities of the instructors. The adolescents had increased knowledge, acquired behavioural skills, and changed certain behaviors. The SRHR-related benefits extended to their siblings and friends. |
| Introduction | | | |  |
| Background/rationale | 2 | Explain the scientific background and rationale for the investigation being reported | 3,4 | An estimated 15% of Nigeria’s 216.7 million population are between the ages of 10 and 24 years [7] with the absolute number of Nigerian youths projected to exceed fifty-seven million by 2025 [8]. The factors responsible for the poor health status of adolescents and young people in Nigeria include inadequate access to health information and services, poverty, as well as inequitable gender norms [9]. A systematic review of the effectiveness of HIV/AIDS school-based sexual health education programmes in Nigeria showed that the adolescents had increased knowledge, healthier attitudes and practiced safer sexual health behaviour [12]. Evidence exists that change in behaviour can be attributed to possession of certain modifiable risk factors namely: information, motivation and behavioral skills. Sexual and reproductive health behaviors are acts influenced by these factors [13,14]. |
| Objectives | 3 | State specific objectives, including any prespecified hypotheses | 4 | The Lagos State Government with funding from UNFPA-UBRAF (2018 to 2020) developed, launched and integrated the CSE program into non-formal educational settings. This study assessed and compared students in CSE exposed and CSE naïve technical and vocational centres in Lagos state to generate a hypothesis regarding its benefits and influence in changing knowledge and attitude. Specifically, to determine and compare comprehensive sexuality education-related knowledge; determine and compare attitudes towards gender norms; and use the IMB model to explore the benefits of teaching and learning about CSE from exposed students and their instructors. The findings will contribute to the body of knowledge regarding this topic among students in non-formal settings and can also be used as an advocacy tool with policy makers. |
| Methods | | | |  |
| Study design | 4 | Present key elements of study design early in the paper | 5 | A mixed-method study design was used made up of a cross-sectional comparative study and a qualitative case study of CSE-exposed technical and vocational centers. |
| Setting | 5 | Describe the setting, locations, and relevant dates, including periods of recruitment, exposure, follow-up, and data collection | 5 | The study population comprised of male and female students attending technical and vocational centers in Lagos State. Data was collected in 2022 |
| Participants | 6 | (*a*) *Cohort study*—Give the eligibility criteria, and the sources and methods of selection of participants. Describe methods of follow-up  *Case-control study*—Give the eligibility criteria, and the sources and methods of case ascertainment and control selection. Give the rationale for the choice of cases and controls  *Cross-sectional study*—Give the eligibility criteria, and the sources and methods of selection of participants | 5,6 | Lagos state has 22 non-formal training institutions (5 technical schools and 17 vocational schools). The courses/trades offered at the vocational centers include Hairdressing & cosmetology; Fashion designing; Textile, Arts & Bead making; Computer studies; Catering; Barbing; Photography. The technical schools offer a wider range of trades such as Bricklaying; Business studies; Furniture making, Plumbing; Welding; Electrical installation; Automobile technology, and Graphic arts. The study population comprised of male and female students attending technical and vocational centers in Lagos State. To be eligible, the student must be between 15 – 24 years of age. Multistage sampling technique was used. Four institutions were randomly selected among the 13 CSE-exposed centers and three among the 9 CSE-naïve centers. Ten courses were randomly selected from the twelve available in technical centers and five from the seven available in the vocational centers. Recruitment in each center was based on its proportionate contribution to the overall sampling frame. Selection of respondents was done using the school’s attendance register |
|  |  | (*b*) *Cohort study*—For matched studies, give matching criteria and number of exposed and unexposed  *Case-control study*—For matched studies, give matching criteria and the number of controls per case |  |  |
| Variables | 7 | Clearly define all outcomes, exposures, predictors, potential confounders, and effect modifiers. Give diagnostic criteria, if applicable | 6 | Knowledge of reproductive organs, condom, contraceptives, HIV & PMTCT; and attitude towards gender norms was assessed. Frequency and percentage as well as summary statistics (mean and standard deviation) were generated. The mean was used for the comparison of the two groups at P<0.05. 5 statements were used to assess perception of gender norms using a 3-point Likert scale. The maximum score based on the most appropriate option is 2marks. Maximum score of 10marks. The higher the score, the more appropriate and relevant the attitude. Overall, individuals with scores > 6 are considered to have good attitude. |
| Data sources/ measurement | 8* | For each variable of interest, give sources of data and details of methods of assessment (measurement). Describe comparability of assessment methods if there is more than one group | 6 | The questionnaire was adapted from the Lagos State sexuality education questionnaire for young persons and administered via Kobotool software on a programmed mobile device through the Open Data Kit (ODK). A mixture of closed and open- ended questions were used to collect the quantitative information. The information was collected by trained data collectors matched by age and gender. Qualitative data was collected using three focus group discussions (FGD) with the students and five key informant interviews with the instructors in the CSE-exposed schools using FGD/KII guide. A mixture of closed and open- ended questions were used to collect the quantitative information. The information was collected by trained data collectors. |
| Bias | 9 | Describe any efforts to address potential sources of bias | 6 | The questionnaires were anonymous, and respondents were assured of the highest level of confidentiality on information given through appropriate data storage and protection. Participation was voluntary and written informed consent was obtained from all participating students above 18years of age and assent from those below 18years. The instructors and staff of the technical colleges and vocational centers were not directly involved in any aspect of data collection and analysis. Data collection took place in the school halls with adequate space to ensure privacy for the participants. |
| Study size | 10 | Explain how the study size was arrived at | 5 | Sample size was calculated using the Cochran formula n = z^2^pq/d^2^ (n is the minimum sample size, z the standard deviation at 95% confidence interval, p of 0.5 and d of 0.5 was used). Sample size of 384 was obtained which was increased to 450 per group. |

Continued on next page

| Quantitative variables | 11 | Explain how quantitative variables were handled in the analyses. If applicable, describe which groupings were chosen and why | 6 | Frequency and percentage as well as summary statistics (mean and standard deviation) were generated. The mean was used for the comparison of the two groups at P<0.05. 5 statements were used to assess perception of gender norms using a 3-point Likert scale. The maximum score based on the most appropriate option is 2marks. Maximum score of 10marks. The higher the score, the more appropriate and relevant the attitude. Overall, individuals with scores > 6 are considered to have good attitude. |
| --- | --- | --- | --- | --- |
| Statistical methods | 12 | (*a*) Describe all statistical methods, including those used to control for confounding |  | The mean was used for the comparison of the two groups at P<0.05. |
|  |  | (*b*) Describe any methods used to examine subgroups and interactions | 6 | Thematic analysis; Intersectionality |
|  |  | (*c*) Explain how missing data were addressed |  | N/A |
|  |  | (*d*) *Cohort study*—If applicable, explain how loss to follow-up was addressed  *Case-control study*—If applicable, explain how matching of cases and controls was addressed  *Cross-sectional study*—If applicable, describe analytical methods taking account of sampling strategy |  | N/A |
|  |  | (*e*) Describe any sensitivity analyses |  | N/A |
| Results | | | | |
| Participants | 13* | (a) Report numbers of individuals at each stage of study—eg numbers potentially eligible, examined for eligibility, confirmed eligible, included in the study, completing follow-up, and analysed | 7 | Nine hundred and thirty-nine students (547 females and 392 males) participated in the study |
|  |  | (b) Give reasons for non-participation at each stage |  | N/A |
|  |  | (c) Consider use of a flow diagram |  | N/A |
| Descriptive data | 14* | (a) Give characteristics of study participants (eg demographic, clinical, social) and information on exposures and potential confounders | 7 | Nine hundred and thirty-nine students (547 females and 392 males) participated in the study. |
|  |  | (b) Indicate number of participants with missing data for each variable of interest |  | N/A |
|  |  | (c) *Cohort study*—Summarise follow-up time (eg, average and total amount) |  |  |
| Outcome data | 15* | *Cohort study*—Report numbers of outcome events or summary measures over time |  |  |
|  |  | *Case-control study—*Report numbers in each exposure category, or summary measures of exposure |  |  |
|  |  | *Cross-sectional study—*Report numbers of outcome events or summary measures | 7-13 | A statistically significant difference was revealed between the exposed and naïve students on the knowledge of five organs namely: Testes; Penis: Scrotum: Uterus and Ovaries while the knowledge of the Vagina and Clitoris was similar. The mean knowledge score for condoms and contraceptives was 1.77+1.472 vs 1.20+1.252; p<0.001). The mean HIV knowledge score was 6.70+1.474 vs 6.34+1.607, p<0.001). A statistically significant difference was observed with regards to PMTCT-related mean knowledge score (2.46+1.044 vs 1.92+1.005; p<0.001). With regards to positive attitude towards gender norms, 15.6% of CSE exposed participants vs 10.8% of CSE naive (p=0.029 had positive attitude).  The theme from the qualitative: Adolescents and instructors’ perception of training-related outcomes achieved. |
| Main results | 16 | (*a*) Give unadjusted estimates and, if applicable, confounder-adjusted estimates and their precision (eg, 95% confidence interval). Make clear which confounders were adjusted for and why they were included |  | N/A |
|  |  | (*b*) Report category boundaries when continuous variables were categorized |  | N/A |
|  |  | (*c*) If relevant, consider translating estimates of relative risk into absolute risk for a meaningful time period |  | N/A |

Continued on next page

| Other analyses | 17 | Report other analyses done—eg analyses of subgroups and interactions, and sensitivity analyses |  | N/A |
| --- | --- | --- | --- | --- |
| Discussion | | | | |
| Key results | 18 | Summarise key results with reference to study objectives | 14-16 | This cross-sectional study carried out among adolescents and young people in non-formal educational settings in Lagos, Nigeria demonstrated significant differences based on exposure to the CSE curriculum. Furthermore, based on the reported changes in knowledge; behaviour skills, adoption of behaviours/lifestyles promoting SRHR and inclusive gender norms we applied the IMB model to improve our understanding of the relationships existing between the CSE curriculum as a tool for teaching and learning by instructors; vulnerable adolescents and young people in non-formal educational settings (Figure 2: Adapted Information-Motivation-Behavioural Skills Model for CSE Sexual Reproductive Health & Rights Behaviour.). We were able to demonstrate the linkages between the SRHR information in the modules, the SRH knowledge acquired and the SRH motivation developed by the adolescents; the connection between these constructs and the life skills component of the curriculum; as well as the pathway for the SRH positive behaviours adopted by the adolescents. |
| Limitations | 19 | Discuss limitations of the study, taking into account sources of potential bias or imprecision. Discuss both direction and magnitude of any potential bias | 16 | In the absence of baseline measurement, it is impossible to categorically state that the CSE curriculum was solely responsible for the difference observed between the two groups of adolescents in our study. |
| Interpretation | 20 | Give a cautious overall interpretation of results considering objectives, limitations, multiplicity of analyses, results from similar studies, and other relevant evidence | 17 | The findings of this study contribute to the body of knowledge on the benefits of CSE for out-of-school young persons and their instructors in SSA. By demonstrating consistently improved SRHR outcomes, the CSE curriculum has the potential to advance the well-being of adolescents and young adults in non-formal settings in countries with similar backgrounds. This research provided a window into how educational interventions can shape perspectives among out-of-school adolescents, young individuals, and instructors in skills-based non-formal settings. When contextualized with broader research, the findings offer both hope and direction for future initiatives aimed at achieving Sustainable Development Goals 3,5 and 10. |
| Generalisability | 21 | Discuss the generalisability (external validity) of the study results |  | The findings may be applicable to young adults in other nonformal settings where trade and skills are acquired in Nigeria and other sub-Saharan African countries. |
| Other information | |  | | |
| Funding | 22 | Give the source of funding and the role of the funders for the present study and, if applicable, for the original study on which the present article is based |  | There was no specific funding for this study |

*Give information separately for cases and controls in case-control studies and, if applicable, for exposed and unexposed groups in cohort and cross-sectional studies.

**Note:** An Explanation and Elaboration article discusses each checklist item and gives methodological background and published examples of transparent reporting. The STROBE checklist is best used in conjunction with this article (freely available on the Web sites of PLoS Medicine at http://www.plosmedicine.org/, Annals of Internal Medicine at http://www.annals.org/, and Epidemiology at http://www.epidem.com/). Information on the STROBE Initiative is available at www.strobe-statement.org.
