# Supplementary material for: The comparison of comprehensive sexuality education knowledge and attitudes in CSE-exposed and CSE-naïve non-formal settings in Lagos State, Nigeria
Source: PLOS Glob Public Health. 2024 Oct 31;4(10):e0003858. doi: 10.1371/journal.pgph.0003858 (PMC11527314; doi:10.1371/journal.pgph.0003858)
Supplement: Attachment — Submitted filename: PGPH-D-24-00960_Review points_WR.docx [file pgph.0003858.s003.docx]

Review points for PGPH-D-24-00960

**Introduction**

Need additional context information including;

1. Please provide detailed information about ‘conservative social norms’ particularly ‘gender norms’ in Nigeria, and its effects on sexuality and reproductive health rights.
2. Please provide demographic information about gender distribution of Nigerian adolescent, and by formal and non-formal educational settings.
3. Please provide HIV/AIDs & STDs epidemiological and unintended pregnancies information among adolescent population in Nigeria with arguments related to gender norms and CSE.

**Material and Methods**

Among 22 non-formal settings including 5 technical (having 12 courses) and 17 vocational centers (having 7 courses),

4. please better clarify the sampling methods to get 10 courses of technical centers and 5 courses of vocational centers;
5. and how to recruit 13 CSE-exposed courses and 9 CSE-naïve courses;
6. and how to recruit CSE-exposed respondents and CSE-naïve respondents, which come out totally 939 respondents for cross-sectional survey.
7. Then state the sub-total numbers of CSE-exposed respondents and CSE-naïve respondents in the starting line of quantitative results.
8. Please also briefly state the scope of issues to be interviewed in FGD in method section.
9. Please recheck the explanation of 3-point Likert scale for assessing perception of gender norms, if it is correct that the maximum score based on the most appropriate option is 2 marks.

**Results**

The qualitative findings need more supportive evidences (quotations) of benefits of CSE, for the following stated issues;

10. Correcting misconception or misunderstanding
11. The sexuality and reproductive health rights that never known before
12. Teaching modalities that help improving understanding
13. Information/skills that help to navigate successful adulthood

**Discussion**

14. Overall comment, please start with the similarity and different results between exposed and naïve groups and address particular outcomes in relation to explain gender norm inequality.
15. Please use IMB model to explain the causal relationship towards transforming negative behaviors regarded gender norms to improve sexuality and reproductive health by CSE such as behavior to prevent HIV/AIDs & STD transmission and infection, unintended pregnancies.
16. Please point out some particular finding issues to tackle for further implementation CSE in non-formal educational settings.
17. Please discuss household or domestic gender inequality that influence sexuality and reproductive health.
18. Please discuss the issue of pedagogy and andragogy in term of adult learning for non-formal setting.

**Conclusion**

19. Please conclude specific findings that support scaling up access to CSE for adolescent in non-formal setting.
