# Supplementary material for: The comparison of comprehensive sexuality education knowledge and attitudes in CSE-exposed and CSE-naïve non-formal settings in Lagos State, Nigeria
Source: PLOS Glob Public Health. 2024 Oct 31;4(10):e0003858. doi: 10.1371/journal.pgph.0003858 (PMC11527314; doi:10.1371/journal.pgph.0003858)
Supplement: Attachment — Submitted filename: RESPONSE TO REVIEWERS COMMENTS SEPTEMBER 2024.docx [file pgph.0003858.s004.docx]

**RESPONSE TO REVIEWER’S COMMENTS PGPH-D-24-00960**

| Reviewer’s comments |  |
| --- | --- |
| Reviewer 1 |  |
| Introduction |  |
| 1. Please provide detailed information about ‘conservative social norms’ particularly ‘gender norms’ in Nigeria, and its effects on sexuality and reproductive health rights. | This is now reflected in the manuscript |
| 2. Please provide demographic information about gender distribution of Nigerian adolescent, and by formal and non-formal educational settings. | This is now reflected in the manuscript |
| 3. Please provide HIV/AIDs & STDs epidemiological and unintended pregnancies information among adolescent population in Nigeria with arguments related to gender norms and CSE | This is now reflected in the manuscript |
| Material and Methods Among 22 non-formal settings including 5 technical (having 12 courses) and 17 vocational centers (having 7 courses), |  |
| 4. please better clarify the sampling methods to get 10 courses of technical centers and 5 courses of vocational centers; | Simple random sampling was used to select 10 courses and 5 courses respectively from the technical and vocational centers. Students attending those courses were eligible for recruitment into the study. |
| 5. and how to recruit 13 CSE-exposed courses and 9 CSE-naïve courses; | In the first stage of sampling, the centers were grouped into CSE naïve and CSE exposed based on whether the students had been taught the CSE curriculum or not. Therefore, we had technical (exposed and naïve); vocational (exposed and naïve). In all there were 13 CSE-exposed centers (technical & vocational) and 9 CSE-naïve centers (technical & vocational). Four centers were randomly selected among the 13 CSE-exposed centers and three among the 9 CSE-naïve centers. The sample size for each institution was based on the proportional contribution of that center to the sampling frame population |
| 6. and how to recruit CSE-exposed respondents and CSE-naïve respondents, which come out totally 939 respondents for cross-sectional survey. | Sample size of 384 was obtained which was increased to 500 per group. |
| 7. Then state the sub-total numbers of CSE-exposed respondents and CSE-naïve respondents in the starting line of quantitative results. | One thousand students participated in the study however 939 entries (456 from CSE exposed respondents and 483 from CSE naïve respondents) could be analysed |
| 8. Please also briefly state the scope of issues to be interviewed in FGD in method section. | The Qualitative aspect of the study explored the benefits of teaching and learning CSE among instructors and students in CSE exposed schools, data was collected using three focus group discussions (FGD) with the students and five key informant interviews with the instructors using FGD/KII guide. |
| 9. Please recheck the explanation of 3-point Likert scale for assessing perception of gender norms, if it is correct that the maximum score based on the most appropriate option is 2 marks. | The desirable response is 2marks, Indifferent is 1mark, undesirable response 0mark |
| Results The qualitative findings need more supportive evidences (quotations) of benefits of CSE, for the following stated issues; |  |
| 10. Correcting misconception or misunderstanding | This is now reflected in the manuscript |
| 11. The sexuality and reproductive health rights that never known before | This is now reflected in the manuscript |
| 12. Teaching modalities that help improving understanding | This is now reflected in the manuscript |
| 13. Information/skills that help to navigate successful adulthood | This is now reflected in the manuscript |
| Discussion |  |
| 14. Overall comment, please start with the similarity and different results between exposed and naïve groups and address particular outcomes in relation to explain gender norm inequality. | The qualitative aspect of the study took place in CSE exposed schools. There is no verbatim from CSE naïve schools for comparison.  The quantitative data was compared not qualitative |
| 15. Please use IMB model to explain the causal relationship towards transforming negative behaviors regarded gender norms to improve sexuality and reproductive health by CSE such as behavior to prevent HIV/AIDs & STD transmission and infection, unintended pregnancies. | The section has been rewritten however, the study design did not permit us to discuss causality but to show linkages. |
| 16. Please point out some particular finding issues to tackle for further implementation CSE in non-formal educational settings. | This is now reflected in the manuscript |
| 17. Please discuss household or domestic gender inequality that influence sexuality and reproductive health. | This is now reflected in the manuscript |
| 18. Please discuss the issue of pedagogy and andragogy in term of adult learning for non-formal setting. | This is now reflected in the manuscript |
| Conclusion 19. Please conclude specific findings that support scaling up access to CSE for adolescent in non-formal setting. | This is now reflected in the manuscript |
| Reviewer 2 |  |
| The main objective of the study was to compare the sexual reproductive health and rights related knowledge and attitudes towards inclusive gender norms among students in comprehensive sexuality education (CSE) exposed and naïve technical and vocational centers in Lagos state, Nigeria. It also explored the benefits of teaching and learning about CSE from CSE-exposed students and their instructors. The strength of the manuscript is to shed the light to the knowledge and attitudes related to sexual and reproductive health, including gender norms among students in technical and vocational centers in Lagos state, Nigeria. These findings could help to inform improvement of CSE curriculum as well as implementation of other interventions to address the gap in non-formal educational settings in the Lagos state, Nigeria. The manuscript also compares the knowledge and attitudes of CSE in intervention and non-intervention centers at the endline, however, the cross-sectional study design, as authors rightly note, is not an ideal design to demonstrate improvement, attributable to intervention; It does not explore potential compounding factors, including those in which CSE exposed and CSE-Naïve student were statistically different (such as sex, marital status and level of education) and does not allow controlled assessment of expected study outcomes before and after the intervention (attributable difference). Below, please find key suggestions to improve the manuscript. The lines of the manuscript are not numbered per PLOS LaTeX template. It is therefore difficult to provide detailed review of the manuscript. In the review, I will therefore highlight the feedback per sections without indicating lines Abstract | |
| 1. Based on the focus group interviews and key informant interviews in CSE-exposed centers, the study concludes that “CSE training benefits not only the students but also their instructors, who acquire new teaching skills and improve their parenting skills”. It is important to highlight that these claims are made based on self-reported data. | Noted and incorporated into the section |
| 2. While the conclusion about importance to integrate CSE into non-formal education settings could be made, the claim that “CSE-exposed schools had statistically significantly higher SRHR-related knowledge scores and positive attitudes towards inclusive gender norms” should be revised to clarify that the difference in knowledge and attitudes among CES exposed and naïve centers, although significant, was only measured at the endline and could not be attributed to the intervention without the knowledge of baseline data and students’ previous exposure to CSE curriculum in schools. | This is now reflected in the manuscript |
| 3. Similarly, without the clear evidence, the abstract conclusion should be revised as: “The study findings suggest that the integration of CSE into non-formal educational settings could improve SRHR-related knowledge among young people, promote inclusive gender norms and contribute to improve SRH outcomes and related SDGs in Nigeria”. | This has been reflected in the updated abstract. |
| Introduction 1. The manuscript describes the evidence related to The Family Life and HIV Education (FLHE) curriculum is the school-based settings in Nigeria and related systematic review, that highlights the effectiveness of FLHE program in improving knowledge, attitude and safer sexual health behaviors among youths. For actionability and scale up, it would be helpful to compare the content of the FLSE curriculum to integrated CSE program (intervention) for non-formal education settings. Furthermore, comparative description of these two curricula (key themes) and the six modules CSE program would help to better understand the program results on one hand and identify actionable recommendations to further improve the curriculum in the areas, where CSE-exposed students did not show improved knowledge and/or attitude. | The FLHE curriculum (2^nd^ edition) is one of the documents used during the desk review process for the development of the CSE curriculum (1^st^ edition). All the topics in the FLHE are in the CSE curriculum and vice versa however the CSE is arranged in a modular format for integration of thematic areas and logical flow as well ease of teaching and learning. With regards to scale-up, the 2^nd^ edition of the CSE curriculum is currently in use. This version has more topics, has a workbook and the web version is currently being developed. |
| Materials and Methods: |  |
| 1. Cross-sectional study design, as authors rightly note, is not an ideal design to demonstrate improvement, attributable to intervention, as it does not allow controlled assessment of expected study outcomes both at the baseline and endline (attributable difference) and the influence of compounding factors. | Mentioned in the limitation. This is noted and would be addressed as a limitation  This study generated a hypothesis regarding the ability of the CSE curriculum to make a difference in knowledge, attitude towards inclusive social norms, acquisition of behaviour skills and adoption of positive SRHR behaviours. However, the limitation of this study design is that it does not allow controlled assessment of expected study outcomes both at the baseline and endline (attributable difference) and the influence of compounding factors. |
| 2. Please, provide further information on the composition of focus groups and key informant interviews, particularly their representativeness (number/% of selected CSE study cites) and the method of selection of participants for GFD and KIIs | 3 FGDs were held in 3 schools made up of 10 students in each. The interviewees were recruited by the data collectors during the quantitative data collection. The CSE instructors in 4 schools participated in the in-depth interviews. One school had 2 instructors while the remaining had one each (5 in total) |
| Results |  |
| Quantitative Analysis 1. Given statistically significant difference in sex of study participants between CSE and non-CSE sites, it would be critical to disaggregate study findings by sex and present sex-disaggregated findings for gender norms in two tables (one for boys and one for girls), instead of Table 3 and move Table 3 in the annexes. | Gender norms table disaggregated by sex (3a and 3b) |
| Qualitative Analysis 1. The reflection from Female JSS needs to be checked for consistency. It now reads as” based on this subject well, I had, I now know how to defend myself when I want to be sexually abused…”. | Verbatim has been amended |
| 2. Please, present the KIIs results on page 12 as self-reported claims by instructors (last paragraph of the page) | This is now reflected in the manuscript |
| Discussion: 1. Please, revise the 3rd line of discussion section as: should read as “based on the self-reported change” … | This is now reflected in the manuscript |
| 2. Considering that the life skills and behaviors were self-reported by learners and instructors and could not be objectively assessed and/or compared to non-intervention setting, please revise the 1st paragraph of the discussion as: “We were able to highlight potential linkages between the SRHR information in the modules, the SRH knowledge acquired and the SRH motivation developed by the adolescents; the connection between these constructs and the life skills component of the curriculum; as well as the potential pathway for the SRH positive behaviors reported by the adolescents”. | This is now reflected in the manuscript |
| 3. Suggest using identified need in the 2nd paragraph of page 14 (With an estimated population of 10 million out-of-school adolescents, research and publications focusing on CSE for this population in Nigeria is limited) to promote the curriculum integration in non-formal education settings, but also discuss potential generalizability and relevance of the study findings beyond Lagos state in Nigeria. | Not sure of the amendment requested by the reviewer  By demonstrating consistently improved SRHR outcomes, the CSE curriculum has the potential to advance the well-being of adolescents and young adults in non-formal settings in other states within the country as well as countries with similar backgrounds. |
| 4. Given the fact that there were clear areas of weaknesses in SRHR knowledge and gender-related attitudes in CSE-exposed settings, the manuscript could be significantly improved by suggesting actionable recommendations to further improve the CSE curriculum in respective areas. | However, given that there were clear areas of weaknesses in SRHR knowledge and gender-related attitudes in CSE-exposed settings in this study, a review of the curriculum and teaching methodology is required to identify areas for improvement to increase its effectiveness. |
| 5. As suggested under results section, please, analyze the potential effect of CSE on gender-norms based on sex-disaggregated study results. | Section rewritten |
| 6. The claim that “The Mixed-method design of this study gives additional validity to the findings based on the integration of the results from the quantitative and qualitative aspect” need to be strengthened with appropriate documentation of similar variables from different data sources. Please, add such results in the annexes to back up the claim or delate it. | Deleted |
| 7. Given that the learners were previously exposed to some of the topics during school education, it is important to note that the study did not examine/account for a previous exposure to CSE education among CSE-exposed and CES naïve study participants, as study limitation in the discussion section. | Section rewritten. The limitations are in the conclusion |
| Conclusion: 1. Without the clear evidence and self-reported claims by study participants, the abstract conclusion should be revised as: “The study findings suggest that the integration of CSE into non-formal educational settings could improve SRHR-related knowledge among young people, promote inclusive gender norms and potentially contribute to improved SRH outcomes and related SDGs in Nigeria”. | This is now reflected in the manuscript |
| Figure 1 1. Revise the tile of the figure as: Self-reported knowledge, skills and behaviors among instructors and CSE-exposed students (indicate number of participants in FGs and KIIs). | This is now reflected in the manuscript |
